# Understanding the Health Behaviors of Survivors of Childhood and Young-Adult Cancer: Preliminary Analysis and Model Development

**DOI:** 10.3390/children2020174

**Published:** 2015-05-07

**Authors:** Stefanie C. Vuotto, Mary E. Procidano, Rachel A. Annunziato

**Affiliations:** Department of Psychology, Fordham University, 441 E. Fordham Rd., New York, NY 10458, USA; E-Mails: procidano@fordham.edu (M.E.P.); annunziato@fordham.edu (R.A.A.)

**Keywords:** childhood cancer, survivorship, health behavior, late effects

## Abstract

The current study presents preliminary correlational data used to develop a model depicting the psychosocial pathways that lead to the health behaviors of survivors of childhood and young-adult cancer. Data collected from a sample of 18- to 30-year-old cancer survivors (n = 125) was used to examine the relations among interpersonal support and nonsupport, personal agency, avoidance, depressive symptoms and self-efficacy as they related to health behaviors. The outcome measures examined included tobacco and alcohol use, diet, exercise, sunscreen use, medication compliance and follow-up/screening practices. Correlational analyses revealed a number of significant associations among variables. Results are used to inform the development of a health behavior model. Implications for health promotion and survivorship programming are discussed, as well as directions for future research.

## 1. Introduction

Cancer is the leading cause of disease-related death among children in the United States, and over 15,000 childhood cancer diagnoses surface each year. Yet while incidence climbs, so do 5-year survival rates, soaring from 61% to 84% since 1975 [1]. As a result, the number of childhood cancer survivors in the US has grown considerably to approximately 400,000. The emergence of this new population has resulted in a field devoted entirely to understanding “survivorship”. Survivorship research investigating the aftermath of childhood cancer consistently demonstrates that survivors are at increased risk for a host of post-remission health complications and mortality due to cancer therapy and related secondary cancers [2]. Approximately two-thirds of all pediatric cancer survivors have at least one complication due to their cancer treatment, with one-third of survivors having serious or life-threatening complications [3].

Ideally, pediatric cancer survivors confronted with such health risks would diligently adhere to follow-up treatment guidelines and adopt physician-recommended health behaviors. In fact, research has demonstrated that adolescent and young-adult survivors report an understanding of their increased susceptibility to post-remission health complications, a need to improve health behaviors, and a desire to improve health behaviors [4]. Unfortunately, many do not report actually following such recommendations [5,6]. While the extent to which adolescent and young-adult survivors engage in risky health behaviors (e.g., tobacco and alcohol use) is comparable to their same-age peers [7,8,9], the implications are far more devastating for a population whose threshold for health complications is already lowered due to organ function disturbances from powerful chemotherapy and radiation treatments and/or genetic predisposition [7]. Health behaviors related to screening and prevention (e.g., sun-protection, scheduling and receiving outpatient follow-up care) are especially useful for subgroups of pediatric cancer survivors predisposed to certain maladies. Despite the importance of regular screening and prevention for this population, findings from the Childhood Cancer Survivor Study (a multi-institutional study surveying over 20,000 adult survivors) indicate survivors are less likely to receive cancer-related follow-up care or physical examinations as survivors age, and as time since diagnosis increases [10].

In order to understand health behaviors in this population, it is useful to consider a developmental framework. Erikson’s Psychosocial Stage theory predicts that adolescents and young-adults must develop a sense of self and foster the development of a secure personal identity in order to continue along a healthy developmental trajectory and master later developmental tasks [11]. For young cancer survivors, this developmental stage is further complicated by an interruptive adverse life event, demanding the attention and focus of resources that might have been directed otherwise (e.g., fostering the young person’s developing sense of self). Conflicts related to personal identity, life meaning or achievement may resurface after the completion of treatment [12], and may be manifested in the form of personal neglect and poor self-management; both barriers to health behavior promotion.

Self-efficacy has been recognized as a key component in cultivating health-promoting behaviors. To better understand people’s decisions to engage in health behaviors, researchers have examined self-efficacy as it relates to health behaviors in the general population [13,14] and among patients with chronic illness [15]. Self-efficacy modifies health behavior via “outcome expectations” (beliefs about whether some behavior will lead to some outcome) and “efficacy expectations” (beliefs about how capable an individual is to perform some behavior) [16]. In gauging the difficulty of health-related tasks and individuals’ abilities to perform those tasks, one can estimate whether investment in those tasks is worthwhile. Additionally, the expected benefits associated with the outcome that results from pursuing a given health behavior play a crucial role in both the initial motivation and decision to adopt that health behavior [17]. Research supporting the association between self-efficacy and health behaviors such as physical activity and exercise [18], pain coping and management [19] and adjustment to rheumatoid arthritis [15,20] has mainstreamed the inclusion of self-efficacy in health psychology research, and has demonstrated that interventions intended to modify self-efficacy can also modify health behavior [13,14].

The extant literature concerning health behaviors among childhood cancer survivors largely—and importantly—report prevalence estimates, treatment-related associations, risk factors, implications and intervention efficacy, but are either limited to the constructs available in larger cohort studies or do not utilize a “bottom-up” approach in conceptual model development. Utilizing a “bottom-up” approach allows progression from individual-level elements (e.g., person-centered traits such as personal agency) to a more holistic understanding of the target behaviors during the process of model construction. A model elucidating the psychosocial pathways to survivors’ health behavior is needed to inform future applied research and intervention design. In the context of young-adult cancer survivorship, one might expect a number of psychosocial factors (including self-efficacy) to contribute to survivors’ health-related decisions and behaviors. The current paper identifies four factors that we expect will demonstrate a robust association with positive health behaviors. As this is a preliminary study, the primary aim is to uncover any existing associations among the variables, with a secondary aim of providing *a priori* rationale for the development of a health behavior model.

The first predictive factor, social support, is rooted in the belief that self-efficacy and positive health beliefs about health behaviors are fostered with the support and encouragement of family or friends. In fact, “verbal persuasion”, rooted in social support and encouragement, is identified as one of four sources said to influence or shape efficacy expectations [16]. Nonsupport (or support perceived as intrusive) is also included, as it has been empirically linked to poor health and well-being outcomes [21]. The second factor, personal agency, is a construct capturing personal identity and autonomy. Beliefs about personal mastery and the development of personal agency promote a sense of control that, in the cancer survivor population, may affect health behaviors and treatment compliance [22]. The third factor, depressive symptoms, has been associated with health behavior outcomes in the general population [23,24,25]. It has been suggested that in the context of adopting positive health behaviors, negative mood affects self-efficacy, which in turn creates the illusion of failure or inefficacy and perpetuates the cycle, increasing despondency [26]. In the context of young-adult cancer survivors, this is especially concerning, as survivors are significantly more likely than sibling controls to report symptoms of depression [27]. A fourth factor, avoidance, is hypothesized to predict survivors’ health behaviors as well. In considering the association between depressive symptoms and health behaviors, it is reasonable to suspect that trauma-related constructs may also affect the likelihood of cancer survivors’ engaging in health behaviors. From an empirical standpoint, avoidance behaviors have been associated with higher perceived stress and anxiety and reduced health-related quality of life [28,29]. Individuals who adopt avoidance behaviors are less likely to directly acknowledge and address perceived stress, potentially resulting in poorer cognitive and physical functioning [30]. For young-adult cancer survivors exhibiting symptoms of avoidance, failure to acknowledge or address perceived stress (e.g., late-effects) could entail evading recommended health behaviors and/or follow-up care and screening. Using the Impact of Event Scale (IES) [31] which defines avoidance as “active attempts to suppress thinking about a stressful event or circumstance” (e.g., “I tried not to think about it”), the current study investigated the relationship between avoidance and health behaviors.

## 2. Methods Section

### 2.1. Participants

Young-adult cancer survivors were recruited via e-mail for the current study using two organizational listservs; the first belonging to a nationwide young-adult cancer survivorship nonprofit organization and the second belonging to a long-term follow-up clinic of a major medical center in New York. Invitational e-mails were sent to e-mail addresses on these listservs from the programs themselves, and interested participants contacted the principal investigator. The listservs of both survivor organizations together comprised of over 7000 e-mail addresses. Notably, any number of these e-mail addresses may have been expired, unused, or duplicates belonging to non-members and/or belonging to individuals who did not meet the eligibility criteria for the study. Unfortunately, there is no way to ascertain the percentage of eligible participants who actually participated in the study. One hundred seventy-two (172) individuals contacted the principal investigator expressing interest in study participation. Of 172 interested persons, 125 completed informed consent procedures and the online survey.

Eligibility requirements for the current investigation stipulated that participants must be English-speaking, literate survivors of childhood cancer who (a) were between the ages of 18 and 30; (b) were in remission (e.g., off active treatment) for at least one year; (c) had access to a device equipped with internet access (e.g., computer, tablet, smart phone) and (d) were competent to provide informed consent. Competency was determined based on years of education (minimum of 8). Informed consent demonstrated understanding of the goals of the study, the participant’s understanding of the voluntary nature of participation, and that receipt of program services would not be associated with participation. Incidence or frequency of relapse was not used as eligibility criterion.

### 2.2. Measures

**Demographic Survey.** A survey was developed for the current investigation to collect demographic data. Social demographic variables included age, gender, socioeconomic status, race, ethnicity and marital status. A composite score ranging from 0 to 6 was used to indicate socioeconomic status (SES). This scale was adapted from a home affluence scale based on adolescents’ reports of material conditions in the home as a measure of family SES [32], and included the following six socioeconomic indicators: (a) whether the participants had completed high school (either by obtaining a diploma or GED); (b) whether the home the participant grew up in was rented or owned; (c) whether the participant’s family had access to one or more cars growing up; (d) whether one or both of the participant’s parents attended college; (e) whether the participant, individually, was eligible for free lunch in school; and (f) if the participant lived with a father or stepfather growing up, whether that person was employed. As many young-adults (perhaps especially cancer survivors) in modern times attain financial stability and independently sustain their living costs at older ages, SES items related to socioeconomic backgrounds and upbringing were selected in lieu of household income and employment status. Level of educational attainment was assessed but should be interpreted with caution as participant ages ranged from 18 to 30 years of age, and therefore some participants were too young to have completed college degrees at the time of the study. Data related to health information such as diagnosis, age at diagnosis, time since completion of treatment, time since first diagnosis, duration of treatment, type of treatment(s) received, and number of late effects were also collected. For the purposes of the study, the term *late effect* was defined on the demographic survey as “a side effect of cancer treatment that appears after the completion of treatment”.

**Self-Efficacy.** The New Generalized Self-Efficacy Scale (NGSE) was used to measure self-efficacy. This 8-item questionnaire measures a general sense of perceived self-efficacy, which is one’s estimate of one's overall ability to perform successfully in a wide variety of achievement situations, or to how confident one is that she or he can perform effectively across different tasks and situations [33]. For the current study, and in accordance with theory and the literature on self-efficacy, items have been slightly adapted to reflect participants’ self-efficacy as it specifically related to engaging in positive health behaviors. Though this has not yet been validated for use with a healthcare population, or with young-adults, internal consistency was high (Cronbach’s α = 0.86) and test-retest coefficients indicate stability (*r* = 0.67) with young-adult undergraduate college students after three administrations over the course of approximately 67 days [33]. The 8 items use a five-point Likert-scale (scored 1–5), ranging from “Strongly Disagree” to “Strongly Agree”, and there is maximum possible score of 40.

**Social Support and Nonsupport.** Specific instances of interpersonal support and nonsupport were assessed using the Interpersonal Support and Nonsupport, Short Form (ISNS-S) [34]. The Interpersonal Support and Nonsupport measure (ISNS) is a self-report instrument consisting of 20 items (10 support items, 10 nonsupport items) which features statements about the participant’s experience of support or nonsupport from a particular individual, in the context of coping with a particular stressful experience, or pursuing an important personal goal striving [35]. For the current study, this experience/goal was specifically outlined as participants’ health and health behaviors. Internal consistency reliability estimates average 0.93 for support and 0.90 for nonsupport. The current study used data collected using the ISNS-S, a short form consisting of 6 support items and 7 nonsupport items from the original ISNS measure. These items were chosen based on a specific item-discrimination criterion; each of the support items was found to correlate with a measure of psychological well-being, and each of the nonsupport items to correlate with a measure of distress [36]. The internal-consistency reliabilities are comparable to those for the full scales reported above.

**Personal Agency.** Personal agency was measured using the agency subscale on the Extended version of the Personal Attributes Questionnaire (EPAQ) [37]. The agency subscale of the EPAQ has eight self-report items that are rated on a 5-point bipolar Likert scale, ranging from 1 (“not at all” possessing the attribute) to 5 (“very” much possessing the attribute), for example, “not at all independent” to “very independent.” Internal consistencies for the agency subscale were obtained with samples of patients with chronic illness (Cronbach’s α = 0.70) and college students (Cronbach’s α = 0.74) [38].

**Depressive Symptoms.** Depressive symptoms were measured using the Center for Epidemiologic Studies Depression Scale (CES-D); a short self-report scale with scores ranging from 0 to 60, designed to measure depressive symptoms in the general population. The new scale was tested in household interview surveys and in psychiatric settings. It was found to have very high internal consistency (Cronbach’s α = 0.84–0.90) and an adequate test-retest reliability of 0.51–0.70. Construct validity is good, and was established by patterns of correlations with other self-report measures, by correlations with clinical ratings of depression, and by relationships with other similar variables [35]. The psychometric properties of the CES-D have also been examined with cancer patients (aged 18 years or older), and were found to have good internal consistency (Cronbach’s α > 0.85) for cancer patients and healthy control groups, as well as adequate test-retest reliability in both groups.

**Avoidance.** To measure avoidance, the Impact of Events Scale (IES) was used. This 15-item self-report measure defines avoidance as an active attempt to suppress thinking about a stressful event or circumstance, and is designed to assess current subjective distress for any specific life event [31]. For the current study, items were adapted to reflect avoidance behaviors as they specifically relate to avoiding thoughts about health/health behaviors. Of the 15 items, 7 items measure intrusive symptoms (intrusive thoughts, nightmares, intrusive feelings and imagery), and 8 items measure avoidance symptoms (numbing of responsiveness, avoidance of feelings, situations, ideas). Respondents are asked to rate the items on a 4-point Likert scale according to how often each has occurred in the past 7 days, with responses ranging from 0 (not at all), to 1 (rarely), to 3 (sometimes), to 5 (often). Both the intrusion and avoidance subscales have displayed acceptable reliability (Cronbach’s α of 0.79 and 0.82, respectively), a split-half reliability for the whole scale of 0.86 [33]. The IES has also displayed good content validity, with the initial study reporting a correlation between the IES avoidance and intrusion scales to be 0.41.

**Health Behaviors.** Health behaviors were measured using a version of 2013 National Youth Risk Behavior Survey (YRBS), adapted for use with a young-adult cancer survivor population. This national school-based survey conducted by the Center for Disease Control (CDC) as well as by state and local education and health agencies monitors six categories of priority health-risk behaviors among youth and young-adults: safety, unintentional injuries and violence; tobacco use; alcohol and other drug use; sexual behaviors; unhealthy dietary behaviors; and physical inactivity. Several items were reworded so that they were appropriate for use with an older adolescent and young-adult population (e.g., direct references to high school were removed). To adapt the survey for use with young-adult cancer survivor populations, items pertaining to the healthy behavior practices of young-adult cancer survivors were added. These additional questions were developed based on the health behaviors and practices reported most frequently in studies using CCSS samples [5,6,39,40]. The final domains of health behavior measured included tobacco use, alcohol use, diet, physical activity, self-care (e.g., medication compliance, adherence to follow-up regimens), safety (e.g., while driving), sun safety and sleep. A preliminary factor analysis conducted on the adapted YRBS data (*n* = 125) indicated a three-factor solution for all reported health behaviors, with the three factors being tobacco use (Cronbach’s α = 0.79), unhealthy lifestyle behaviors (reverse-coded items related to diet, exercise, sunscreen application, and medication adherence; Cronbach’s α = 0.69), and risk/neglectful behaviors (items related to safe driving habits, adherence to long-term follow-up regimens, and sunburn; Cronbach’s α = 0.69).

### 2.3. Procedure

Participants who expressed interest in partaking in the current study via e-mail were sent an e-mail (a) confirming participants’ ability to meet eligibility criteria; (b) providing instructions for submitting the electronic informed consent form; (c) providing the participant ID number used to verify participation and provide compensation; (d) providing instructions for the participant to complete the survey in a quiet, private space, and to allot a time block of 45 min for the survey; and (e) furnishing the link to the online survey. Participants were given the opportunity to ask questions at this time, via e-mail or telephone. Within 30 days of providing consent, participants completed the online questionnaire which contained scales of self-report measures relating to health behavior practices, self-efficacy, social support and nonsupport, personal agency, depression and avoidance. Upon completion of the survey, each participant was e-mailed a $10 gift card. This study was approved by the Fordham University Institutional Review Board.

### 2.4. Statistical Analysis

In accord with exploratory goals of the current study (e.g., model development), correlation analyses were performed to explore potential associations between study variables. A power analysis was conducted to determine how many subjects would be required in order to achieve a desired power of 0.80 (β = 0.20) at α = 0.05 using correlation analyses, as was planned for the current study. The necessary sample size required to detect a large effect size and to achieve statistical significance with a sample correlation of *r* = 0.3 was 85 participants; with a sample correlation of *r* = 0.5 was 29 participants, and with a sample correlation of *r* = 0.7 was 13 participants.

## 3. Results Section

### 3.1. Sample Descriptors

Participant demographic characteristics are described in Table 1. Participants reported having survived a wide range of cancer diagnosis, including blood cancers, solid tumors and cancers of the brain and central nervous system (see Table 2). Survivors reported having received a variety of cancer treatment modalities, with 93% reporting having received any chemotherapy, 73% receiving any radiation therapy, 62% receiving any surgical treatment, and 15% receiving a bone marrow or stem cell transplant. Over half (53.6%) of the sample reported having at least one long-term late effect as a result of their cancer treatment.

**Table 1:** Demographic characteristics of the sample.

	M	SD	Range
Age (years)	24.09	3.46	18–30
Socioeconomic Status (scale of 0–6)	5.34	0.84	2–6
		*N*	%
Gender			
Female		94	75.2%
Male		31	24.8%
Marital Status			
Single/Never Married		102	81.6%
Married or Domestic Partnership		14	11.2%
Divorced		2	1.6%
Widowed		0	-
Engaged		7	5.6%
Race			
American Indian or Alaskan Native		1	0.8%
Asian		9	7.2%
Black (African-American)		13	10.8%
Black (Caribbean-American)		3	2.4%
Native Hawaiian or Pacific Islander		1	0.8%
White		98	78.4%
Ethnicity			
Non-Hispanic		109	87.2%
Hispanic		16	12.8%
Level of Education			
Grade School		0	-
Some High School		1	0.8%
GED		1	0.8%
High School Diploma		3	2.4%
Some College		41	32.8%
Associate’s Degree		8	6.4%
Bachelor’s Degree		50	40%
Graduate Degree		21	16.8%

**Table 2:** Health and survivorship characteristics of the sample.

	M	SD	Range
Time Since First Diagnosis (years)	8.29	5.69	2–29
Time Since Last Cancer Treatment (years)	6.61	5.41	1–26
Age at First Diagnosis	15.8	6.7	1–27
		*N*	%
Number of Known Late Effects			
Didn’t know		20	16
None		38	30.4
One		28	22.4
Two		20	16
Three or more		19	15.2
Primary Diagnosis			
Leukemia		27	21.6
Brain/CNS		15	12
Neuroblastoma		2	1.6
Wilm’s Tumor		5	4
Lymphoma		41	32.8
Rhabdomyosarcoma		1	0.8
Retinoblastoma		1	0.8
Osteosarcoma/Ewing’s Sarcoma		12	9.6
Non-CNS/Other		21	16.8

### 3.2. Correlation Analysis

Bivariate correlations between all study variables were examined in order to (a) assess the extent of multicollinearity among the data and (b) uncover any potential relationships between variables which many inform model development (see Table 3). Six independent variables (social support, nonsupport, personal agency, depressive symptoms, avoidance and self-efficacy) and three outcome variables (tobacco use, unhealthy lifestyle behaviors and risk/neglectful behaviors) were correlated in expected directions and magnitudes, falling in the low (e.g., *r* = 0.2–0.3) and moderate (e.g., *r* = 0.3–0.5) ranges [41] and therefore failing to warrant variable exclusion or further investigation of multicollinearity (see Table 3).

**Table 3:** Correlations among study variables.

Variable *(Measure)*	1	2	3	4	5	6	7	8	9
1. Support *(ISNS-S)*	-								
2. Nonsupport *(ISNS-S)*	−0.04	-							
3. Personal Agency *(EPAQ)*	0.28 *	−0.05	-						
4. Depressive Symptoms *(CES-D)*	−0.27 *	0.35 *	−0.54 *	-					
5. Avoidance *(IES)*	0.10	−0.05	−0.06	0.35 *	-				
6. Self-Efficacy *(NGSE)*	0.37 *	0.05	0.40 *	−0.33 *	−0.08	-			
7. Tobacco Use	−0.02	0.05	0.10	−0.03	0.14	0.07	-		
8. Unhealthy Lifestyle Behaviors	−0.21 *	0.01	−0.26 *	0.29 *	−0.03	−0.29 *	−0.08	-	
9. Risk/Neglectful Behaviors	−0.10	−0.02	0.06	−0.04	−0.02	−0.07	0.26 *	0.01	-

* *p* < 0.01 (two-tailed).

Among the three outcome variables, one small positive correlation (*r* = 0.26, *p* < 0.01) was found between tobacco use and risk/neglectful behaviors. Among the six independent variables, there were significant, positive relationships between support and personal agency (*r* = 0.28, *p* < 0.01), support and self-efficacy (*r* = 0.37, *p* < 0.01), and personal agency and self-efficacy (*r* = 0.40, *p* < 0.01). There were significant negative relationships between depressive symptoms and the following variables: support (*r* = −0.27, *p* < 0.01), personal agency (*r* = −0.54, *p* < 0.01) and self-efficacy (*r* = −0.33, *p* < 0.01). Significant positive relationships were observed between depressive symptoms and nonsupport (*r* = 0.35, *p* < 0.01) and depressive symptoms and avoidance (*r* = 0.35, *p* < 0.01). Regarding basic associations between independent and outcome variables, support (*r* = −0.21, *p* < 0.01), personal agency (*r* = −0.26, *p* < 0.01) and self-efficacy (*r* = −0.29, *p* < 0.01) were all significantly inversely related to unhealthy lifestyle behaviors, while depressive symptoms (*r* = 0.29, *p* < 0.01) were significantly positively related to unhealthy lifestyle behaviors.

## 4. Discussion/Conclusions

The primary objective of this study was to explore the association between *a priori* psychosocial factors and young-adult cancer survivors’ health behaviors. By examining the associations between self-efficacy, interpersonal support and nonsupport, personal agency, depressive symptoms and avoidance as they relate to each other and to various health behaviors, the current study attempted to utilize a “bottom-up” approach in laying the foundation upon which health behavior interventions may be built.

### 4.1. Observed Correlations among Independent Variables

A number of study variables demonstrated significant associations with one another, many of which are supported in the literature. Consistent with the literature on self-efficacy, self-efficacy was significantly correlated with interpersonal support (*r* = 0.370) and negatively associated with depressive symptoms (*r* = –0.328). For example, research has demonstrated that adding social network or social support factors to modified versions of the Health Behavior Model enhanced explained variance in the models [42,43]. Other studies have shown cancer survivors’ self-efficacy for specific health behaviors such as physical activity and adherence to exercise regimens to be impacted by depressive symptoms like pain and fatigue [44,45]. Perkins *et al.* found breast cancer survivors’ depressive symptoms to be significantly associated with self-efficacy for physical activity [46]. To an extent, the inverse relationship between self-efficacy and depressive symptoms can be explained when one considers the emotional implications of low self-efficacy. Participants who felt they lacked control over their health or health behaviors may be more likely to experience symptoms of anxiety and depression [47], possibly resulting in poorer health outcomes and further reduced efforts to engage in health behavior change. A medium-to-large sized correlation between self-efficacy and personal agency (*r* = 0.400) was also observed. According to Bandura, mechanisms of personal agency serve as primary contributors to individuals’ psychological functioning, with self-efficacy perhaps being the most central and pervasive agency mechanism humans possess [48]. In this regard, self-efficacy and personal agency are seen as separate but related entities, profoundly contributing to psychological functioning. Given that participants in the current study demonstrated average to high-average beliefs about their ability to exercise control over their health behaviors, and given that they generally endorsed EPAQ items that skewed towards agentic qualities like competitiveness, independence, perseverance, and self-confidence, one might expect their beliefs in their capability to master a given behavior (e.g., self-efficacy) to be associated with their person-centered qualities related to autonomy and self-sufficiency (e.g., personal agency).

For the current study, participants were asked to assess interpersonal nonsupport to the extent that sources of nonsupport impeded or hindered their health behaviors. While studies have examined the harmful effects of *lack of* support, the literature on nonsupport (e.g., “support” that is experienced as intrusive or controlling to the receiver) is quite scarce. Depressive symptoms were significantly associated with interpersonal support (*r* = −0.267) and nonsupport (*r* = 0.354). The inverse relationship between depressive symptoms and interpersonal support is somewhat expected and has been supported in health behavior literature. For example, in a study examining the relationship between depression, support and health behaviors in a sample of international college students, the number of depressive symptoms was negatively correlated with social support in both men (*r* = −0.220) and women (*r* = −0.20) [49].

A novel finding of this study is the relationship between depression and nonsupport. Survivors who perceive the “support” behaviors of family or friends to be diminishing of their personal autonomy may feel smothered by their “support” networks, and be at risk for stifled identity development or depressed mood. The relationship between nonsupport and depression is not terribly different from the way “expressed emotion” (e.g., the degree to which relatives or caregivers display critical or emotionally over-involved attitudes about an individual’s health) has been documented to negatively influence the course of illness in schizophrenic patients [50,51]. Young cancer survivors who live with or in close proximity to relatives whose communication styles are characterized by overprotectiveness, criticism, or excessive emotional involvement may experience these “support” systems as sources of stress and subsequently develop feelings of sadness or despondency. Of course, given the nature of correlations, one must also consider whether the behaviors of others (perceived as nonsupportive by survivors) are actually elicited responses to the burdens and frustrations associated with living with depressed persons. Either way, this relationship draws attention to a truth that may not be intuitive to the families and support networks of cancer survivors: that ostensibly supportive behaviors may not be always be construed as such, and that families should seek to better understand the ways in which their support behaviors can truly be supportive.

Correlations were observed between depressive symptoms and personal agency (*r* = −0.536) and between personal agency and interpersonal support (*r* = 0.283). Individuals who receive social support (and perceive the support to be supportive) may also be more likely to acquire social resources, master essential developmental tasks and move forward along healthy developmental trajectories. The adjustment outcomes for such individuals likely resemble attributes of personal agency such as self-confidence and independence. Cancer survivors’ interrupted psychological, emotional, social or sexual development (by virtue of nonsupport or other adversity) may hinder the development of a sense of personal agency and result in self-perceptions characterized by lack of self-confidence and dependency, which ultimately manifest as depressive symptoms.

A final relationship observed among predicting variables in the present study was that between depressive symptoms and avoidance (*r* = 0.349). As mentioned previously, an *a priori* conceptualization of avoidance was used to assess avoidance in the current study: avoidance was included as a symptom of anxiety brought about by worry over one’s health and health behaviors. In this sense, survivors might utilize avoidance as a coping strategy or defensive mechanism in response to threatening thoughts about their health or health behaviors. Avoidant coping has been demonstrated in the literature to be associated with poorer health [29], lower mental health-related quality of life and perceived stress [30].

### 4.2. Observed Correlations among Independent and Outcome Variables

Several noteworthy correlations between independent and outcome variables emerged as well, such as the correlation between unhealthy lifestyle behaviors and depressive symptoms (*r* = 0.293). The relationship between depression and health behavior has been supported by research conducted among the general population [23] and among young-adults [49]. Studies examining this relationship among childhood cancer survivors have indicated a bidirectional relationship exists between health behaviors and depression, as poor psychological functioning can lead to adverse health outcomes, and vice versa. Mulrooney *et al.* compared a sample of 1897 long-term survivors of childhood cancer to 326 siblings and found a significant association between increased fatigue and depression among survivors, who were significantly more fatigued than sibling comparison subjects [52]. A study of 1101 survivors of CNS tumors found that survivors who rated their own health status as “poor” also reported more symptoms of depression, anxiety and somatic distress [53]. As depressive symptoms can include changes in appetite and diminished motivation, it is not surprising that the current sample demonstrated inadequate levels of healthy eating and physical activity.

The association between personal agency and unhealthy lifestyle behaviors (*r* = −0.264) may be best understood by considering the associations between personal agency and depressive symptoms (*r* = −0.536) and unhealthy lifestyle behaviors and depressive symptoms (*r* = 0.293). Persons with lower scores on agentic qualities (e.g., self-confidence, perseverance, easy decision-making, independence, superiority, competitiveness, *etc.*) may be more prone to experiencing depressive symptoms, and hence more likely to report poorer health behaviors.

The small correlation between support and unhealthy lifestyle behaviors (*r* = −0.212) in the current study is supported by the relationship between support and depressive symptoms (*r* = −0.267) and may be interpreted as such: persons with higher levels of interpersonal support are less likely to exhibit symptoms of depression, and are therefore more likely to engage in healthy lifestyle behaviors. This has been demonstrated in research on support and young-adult cancer *patient* health behaviors, and research has suggested that social support systems characterized by less restrictive and more balanced communication styles improve treatment adherence among adolescent and young-adult cancer patients [54]. Similarly, Burtow *et al.* reported that the degree of openness among family relationships and number and quality of social support resources predicted treatment adherence in young-adult cancer patients [55]. We might expect to see the same relationship between support and healthy adherence behaviors among young-adult survivors. With regard to support and other health behaviors (e.g., diet, exercise, sunscreen application and sleep), an exhaustive search of the literature failed to uncover studies that might support this relationship. It is not unreasonable, however, to postulate that support might reduce uncertainties about and enhance feelings of personal control over one’s health or health behavior. Of course, the capacity of cancer survivors to seek out and utilize interpersonal support from within their social networks might be dependent on other considerations, like their health. For example, immunosuppressed cancer survivors who must maintain sterile, restrictive environments may lack the chance to engage in social support networks or procure interpersonal support, suggesting a possible bidirectional relationship between health/mental health and support.

Finally, a negative correlation was observed between self-efficacy and unhealthy lifestyle behaviors (*r* = −0.291), suggesting that survivors with higher scores for health behavior-specific self-efficacy practiced healthier lifestyle behaviors. Self-efficacy, or a person’s belief that a preventive behavior will achieve a health goal [56], has been widely-studied and implicated in health-behavior change research. Research examining the link between self-efficacy and health behavior has been demonstrated in both the general population [13,15,57,58,59,60] and among those with chronic illness [61,62,63]. For young-adult survivors, it is probable that self-efficacy affects the intention to change behavior, as well as behavior change itself.

### 4.3. Model Development

**Figure 1:**
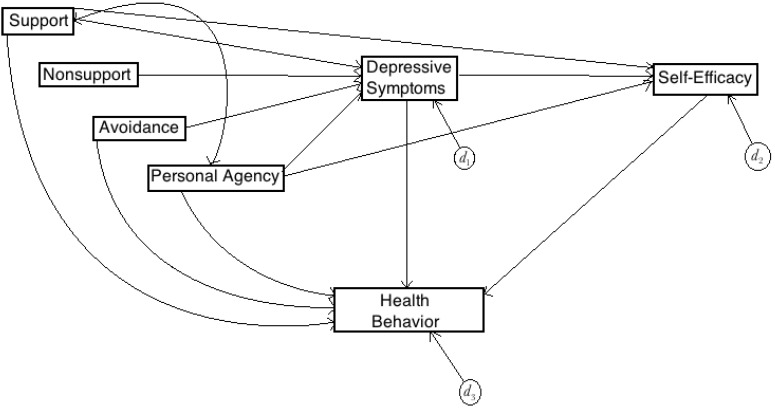
Proposed Model.

After careful consideration of and based on the observed correlations between study variables and the theoretical and conceptual relations that likely define those correlations, a causal model elucidating the psychosocial factors related to young-adult survivor health behavior was constructed (see Figure 1). In the proposed model, symptoms of depression mediate the effects of support, nonsupport, avoidance and personal agency on health behavior, while self-efficacy mediates the effects of support, personal agency and depression on survivors’ health behavior. Although all variables (with the exception of nonsupport) are proposed to have a direct effect on health behavior, depression and self-efficacy emerge as salient predictors of survivor health behaviors, also mediating pathways to health behavior.

### 4.4. Limitations, Implications and Future Directions

There are a number of factors limiting the current study. The self-selecting mode of recruitment introduces the potential for selection bias, and as previously stated, we were unable to ascertain the response rate (percentage of eligible participants who participated in the study). Without access to medical records or patient charts, self-reported information regarding diagnosis, treatment and survivorship status could not be verified. For this reason, with regard to treatment modality, data collection was restricted to “general” treatment modalities (e.g., chemotherapy, radiation, *etc.*) and information regarding specific treatments associated with various impairments (e.g., cranial radiation) was not collected. Additionally, the small sample should be taken into consideration when interpreting findings.

Findings in the current study indicate that resources for health behavior modification programming should primarily focus on survivor “lifestyle” behaviors (e.g., diet, exercise, sunscreen application, medication adherence); a practical and appropriate goal for survivorship programming. A number of important constructs examined in this study such as nonsupport, personal agency and avoidance are lacking in the literature, and should be included in future research as they may be particularly informative in further determining specific avenues of intervention, programming and treatment. The literature also calls for longitudinal studies tracing survivors’ health trajectories into adulthood. Such research might further elucidate the transition from health care on the pediatric side to health care on the adult side as it relates to adherence and health and adjustment outcomes.

## References

[1] Howlader N., Noone A.M., Krapcho M., Garshell J., Miller D., Altekruse S.F., Kosary C.L., Yu M., Ruhl J., Tatalovich Z. SEER Cancer Statistics Review, 1975–2012.

[2] Mertens A.C., Yasui Y., Neglia J.P., Potter J.D., Besbit M.E., Ruccione K., Smithson W.A., Robison L.L. (2007). Late mortality experience in five-year survivors of childhood and adolescent cancer: The childhood cancer survivor study. J. Clin. Oncol..

[3] Oeffinger K.C., Hudson M.M. (2004). Long-term complications following childhood and adolescent cancer: Foundations for providing risk-based health care for survivors. Cancer J. Clin..

[4] Mulhern R.K., Tyc V.L., Phipps S., Crom D., Barclay D., Greenwald C., Hudson M., Thomppson E. (1995). Health-related behaviors of survivors of childhood cancer. Med. Pediatr. Oncol..

[5] Hudson M.M., Tyc V.L., Jayawardene D.A., Gattuso J., Quargnenti A., Greenwald C., Crom D.B., Mason C., Srivastava D.K., Hinds P. (1999). Feasibility of implementing health promotion interventions to improve health-related quality of life. Int. J. Cancer Suppl..

[6] Hudson M.M., Tyc V.L., Srivastava D.K., Gattuso J., Quargnenti A., Crom D.B., Hinds P. (2002). Multi-component behavioral intervention to promote health protective behaviors in childhood cancer survivors: The protect study. Med. Pediatr. Oncol..

[7] Tyc V.L., Hadley W., Crockett G. (2001). Prediction of health behaviors in pediatric cancer survivors. Med. Pediatr. Oncol..

[8] Hollen P.J., Hobbie W.L. (1996). Decision making and risk behaviors of cancer-surviving adolescents and their peers. J. Pediatr. Oncol. Nurs..

[9] Hudson M.M., Findlay S. (2006). Health-risk behaviors and health promotion in adolescent and young adult cancer survivors. Cancer.

[10] Oeffinger K.C., Mertens A.C., Hudson M.M., Gurney J.G., Casillas J., Chen H., Whitton J., Yeazel M., Yasui Y., Robison L.L. (2004). Health care of young adult survivors of childhood cancer: A report from the Childhood Cancer Survivor Study. Ann. Fam. Med..

[11] Erikson E. (1998). Identity, Youth, and Crisis.

[12] Daum A.L., Collins C. (1992). Failure to master early developmental tasks as a predictor of adaptation to cancer in the young adult. Oncol. Nurs. Forum.

[13] Kelly R.B., Zyzanksi S.J., Alemagno S.A. (1991). Prediction of motivation and behavior change following health promotion: Role of health beliefs, social support and self-efficacy. Soc. Sci. Med..

[14] Strecher V.J., DeVellis B.M., Becker M.H., Rosenstock I.M. (1986). The role of self-efficacy in achieving health behavior change. Health Educ. Q..

[15] Taal E., Rasker J.J., Seydel E.R., Wiegman O. (1993). Health status, adherence with health recommendations, self-efficacy and social support in patients with Rheumatoid Arthritis. Patient Educ. Couns..

[16] Bandura A. (1977). Self-efficacy: Toward a unifying theory of behavioral change. Psychol. Rev..

[17] Strecher V.J., Becker M.H., Kirscht J.P., Eraker S.A., Graham-Tomasi R.P. (1985). Psychosocial aspects of changes in cigarette-smoking behavior. Patient Educ. Couns..

[18] Fruin D.J., Pratt C., Owen N. (1992). Protection motivation theory and adolescents’ perceptions of exercise. J. Appl. Soc. Psychol..

[19] Litt M.D. (1988). Self-efficacy and perceived control: Cognitive mediators of pain tolerance. J. Personal. Soc. Psychol..

[20] O’Leary A., Shoor S., Lorig K., Holman H.R. (1988). A cognitive behavioral treatment for rheumatoid arthritis. Health Psychol..

[21] Wethington E., Kessler R.C. (1986). Perceived support, received support, and adjustment to stressful life events. J. Health Soc. Behav..

[22] Rolland J.S. (2005). Cancer and the family: An integrative model. Cancer.

[23] Kobau R., Safran M.A., Zach M.M., Moriarty D.G., Chapman D. (2004). Sad, blue, or depressed days, health behaviors and health-related quality of life, Behavior Risk Factor Surveillance System, 1995–2000. Health Qual. Life Outcomes.

[24] Murphy J.M., Horton N.J., Monson R.R., Laird N.M., Sobol A.M., Leighton A.H. (2003). Cigarette smoking in relation to depression: Historical trends from the Stirling County study. Am. J. Psychiatry.

[25] DiMatteo M.R., Lepper H.S., Croghan T.W. (2000). Depression is a risk factor for noncompliance with medical treatment: Meta-analysis of the effects of anxiety and depression on patient adherence. Arch. Intern. Med..

[26] Bandura A. (1997). Self-Efficacy: The Exercise of Control.

[27] Zebrack B.J., Zeltzer L.K., Whitton J., Mertens A.C., Odom L., Berkow R., Robison L.L. (2002). Psychological outcomes in long-term survivors of childhood leukemia, Hodgkin’s disease, and non-Hodgkin’s lymphoma: A report from the childhood cancer survivor study. Pediatrics.

[28] Blake R., Vandiver T. (1988). The association of health with stressful life changes, social supports, and coping. Fam. Pract. Res..

[29] Sherbourne C., Hays R., Wells K. (1995). Personal and psychosocial risk factors for physical and mental health outcomes and course of depression among depressed patients. J. Consult. Clin. Psychol..

[30] Farley T., Galves A., Dickinson L.M., Perez M.J.D. (2005). Stress, coping, and health: A comparison of Mexican immigrants, Mexican-Americans, and Non-Hispanic Whites. J. Immigr. Health.

[31] Horowitz M., Alvarez W. (1979). Impact of Event Scale: A measure of subjective stress. Psychosom. Med..

[32] Wardle J., Robb K., Johnson F. (2002). Assessing socioeconomic status in adolescents: The validity of a home affluence scale. J. Epidemiol. Community Health.

[33] Chen G., Gully S.M., Eden D. (2001). Validation of a new general self-efficacy scale. Organ. Res. Methods.

[34] Procidano M.E., Pickens I.B., Plantin S.L., Jordan B.L. Brief, Context-Specific Measure of Interpersonal Support and Nonsupport Predicts Adjustment. Proceedings of the Annual Meeting of the American Psychological Association.

[35] Walker-Smith W., Procidano M.E. Support and nonsupport in the context of personal strivings. Proceedings of the Annual Meeting of the American Psychological Association.

[36] Procidano M.E. (1994). The Nature of Interpersonal Support and Nonsupport. Ph.D. Thesis.

[37] Spence J.T., Helmreich R.L., Holahan C.K. (1979). Negative and positive components of psychological masculinity and femininity and their relationships to self‑reports of neurotic and acting-out behaviors. J. Pers. Soc. Psychol..

[38] Helmreich R.L., Spence J.T., Wilhelm J.A. (1981). A psychometric analysis of the Personal Attributes Questionnaire. Sex Roles.

[39] Hudson M.M. (2005). A model for care across the cancer continuum. Cancer.

[40] Robison L., Green D.M., Hudson M.M., Meadows A.T., Mertens A.C., Packer R.J., Sklar C.A., Strong L.C., Yasui Y., Zeltzer L.K. (2005). Long-term outcomes of adult survivors of childhood cancer. Cancer.

[41] Cohen J. (1988). Statistical Power Analysis for the Behavioral Sciences.

[42] Becker M.H., Maiman L.A. (1975). Sociobehavioral determinants of compliance with health and medical care recommendations. Med. Care.

[43] Langlie J.K. (1977). Social networks, health beliefs, and preventive health behavior. J. Health Soc. Behav..

[44] Deimling G.T., Bowman K.F., Wagner I.J. (2007). The effects of cancer-related pain and fatigue on functioning of older adult long-term cancer survivors. Cancer Nurs..

[45] Young K.E., White C.A. (2006). The prevalence and moderators of fatigue in people who have been successfully treated for cancer. J. Psychosom. Res..

[46] Perkins S., Jenkins L.S. (1998). Self-efficacy expectation, behavior performance, and mood status in early recovery from percutaneous transluminal coronary angioplasty. Heart Lung.

[47] Bandura A. (1986). Social Foundations of Thought and Action: A Social Cognitive Theory.

[48] Bandura A. (1989). Perceived Self-Efficacy in the Exercise of Personal Agency.

[49] Allgower A., Wardle J., Steptoe A. (2001). Depressive symptoms, social support and personal health behaviors in young men and women. Health Psychol..

[50] Hooley J.M. (2004). Do psychiatric patients do better clinically if they live with certain kinds of families?. Curr. Dir. Psychol. Sci..

[51] Leff J., Vaughn C. (1985). Expressed Emotion in Families.

[52] Mulrooney D.A., Ness K.K., Neglia J.P., Whitton J.A., Green D.M., Zeltzer L.K., Robison L.L., Mertens A.C. (2008). Fatigue and sleep disturbance in adult survivors of childhood cancer: A report from the childhood cancer survivor study (CCSS). Sleep.

[53] Zebrack B.J., Gurney J.G., Oeffinger K., Whitton J., Packer R.J., Mertens A., Turk N., Castleberry R., Dreyer Z., Robison L.L. (2004). Psychological outcomes in long-term survivors of childhood brain cancer: A report from the childhood cancer survivor study. J. Clin. Oncol..

[54] Kyngas H. (2000). Compliance of adolescents with chronic disease. J. Clin. Nurs..

[55] Burtow P., Palmer S., Pai A., Goodenough B., Luckett T., King M. (2010). Review of adherence-related issues in adults and young adults with cancer. J. Clin. Oncol..

[56] Petosa R., Jackson K. (1991). Using the health belief model to predict safer sex intentions among adolescents. Health Educ. Q..

[57] Norman P., Hoyle S. (2004). The theory of planned behaviour and breast self-examination: Distinguishing between perceived control and self-efficacy. J. Appl. Soc. Psychol..

[58] Armitage C., Conner M. (1999). The theory of planned behaviour: Assessment of predictive validity and “perceived control”. Br. J. Soc. Psychol..

[59] Giles M., McClenahan C., Caims E., Mallet J. (2004). An application of the Theory of Planned Behaviour to blood donation: The importance of self-efficacy. Health Educ. Res..

[60] Terry D.J., O’Leary J.E. (1995). The theory of planned behaviour: The effects of perceived behavioural control and self-efficacy. Br. J. Soc. Psychol..

[61] Lorig K.R., Mazonson P.D., Holman H.R. (1993). Evidence suggesting that health education for self-management in patients with chronic arthritis has sustained health benefits while reducing health care costs. Arthritis Rheum..

[62] Merluzzi T.V., Martinez Sanchez M.A. (1997). Assessment of self-efficacy and coping with cancer: Development and validation of the Cancer Behavior Inventory. Health Psychol..

[63] Marks R., Allegrante J.P. (2005). A review and synthesis of research evidence for self-efficacy-enhancing interventions for reducing chronic disability: Implications for health Education Practice (Part II). Health Promot. Pract..

